# A Qualitative Assessment among Personnel Working in Community-Led Development Program Settings Regarding Sexualized Substance Use

**DOI:** 10.3390/brainsci12111590

**Published:** 2022-11-21

**Authors:** Binata Marik, Nupur Mahajan, Rohit Sarkar, Rochana Mitra, Rajiv Dua, Sumit Aggarwal

**Affiliations:** 1 Division of Epidemiology and Communicable Diseases (ECD), Indian Council of Medical Research (ICMR)-Headquarters, New Delhi 110029, India; 2 India HIV/AIDS Alliance, Zuari House, 6, Kailash Colony, Extension, New Delhi 110048, India

**Keywords:** community health mobilizers (CHMs), formative research, human immunodeficiency virus (HIV), in-depth interviews (IDIs), sexually transmitted infections (STIs), transgender (TG) persons

## Abstract

Sexualized substance use (SSU) is the practice of psychotropic substance use before or during sex to increase sexual pleasure. The growing use of SSU has a strong association with sexually transmitted infections (STIs). Community health mobilizers (CHMs) are agents who assist in decreasing the global burden of disease in the communities they serve. They work as unit managers, counselors, or field workers. The managers and counselors have a minimum of a bachelor’s degree, and field workers have a minimum of a higher secondary education. This study aimed to qualitatively assess the knowledge gaps regarding SSU among CHMs. In-depth interviews (IDIs) were conducted in New Delhi, India with nineteen CHMs. Majority of the CHMs were men (*n* = 9, 47%) followed by transgender (TG) persons (TG females *n* = 5, 26.3%; TG males *n* = 1, 5.2%), and women (*n* = 4, 21.1%). Knowledge gaps were identified among the CHMs regarding different types of sexualized substances, drug procurement, human immunodeficiency virus (HIV) infection prevention, and complex health issues associated with SSU. It suggested the need for periodic workshops and training for upgradation of existing knowledge and practices among the CHMs. This formative research may help social scientists to develop protocols for conducting multi-centric, community-based studies across the country for further validation and exploration.

## 1. Introduction

The use of substances, including alcohol and psychotropic drugs, has a long history and is still a prominent social issue globally [1]. The use of addictive substances might be legal, socially acceptable, or even religious; however, it causes pathological alterations in the brain. Substance users who have developed pathological adaptations are less receptive to interpersonal and social interactions and are more susceptible to compulsive drug seeking and usage [2]. *The Diagnostic and Statistical Manual of Mental Disorders*, *Fifth Edition* (*DSM-V*) recognizes that a variety of substances can contribute to substance-related disorders including alcohol, caffeine, cannabis, hallucinogens, inhalants, opioids, sedatives, hypnotics, stimulants, cigarettes, and anxiolytics [3].

Sexualized substance use (SSU) is the use of recreational substances for the purpose of promoting sexual engagement. Chemsex is considered a subset of SSU, which is commonly defined as the use of specific drugs (methamphetamine, mephedrone, gamma-hydroxybutyrate (GHB/GBL), ketamine and cocaine) before or during sexual intercourse. Chemsex is common among homosexuals, bisexuals, and other men who have sex with men (MSM). According to qualitative research, MSM frequently engaged in SSU/chemsex because they believed that psychoactive chemicals may boost arousal and stamina, enabling prolonged sex sessions. Other reasons included the following: overcoming lack of confidence, increasing the emotional bond with sex partners, and managing stress [4]. To get a greater rush, methamphetamine or mephedrone can also be injected. This is a high-risk behavior that can result in HIV and hepatitis C transmission using shared injecting equipment [5]. Additionally, SSU is also known to have a role in survival sex practices, which includes the selling of sex for subsistence needs such as shelter, food, drugs, or money. A positive association between co-administration of cocaine and methamphetamine with an increased likelihood of involvement in survival sex work was observed in a study among female sex workers [6].

To understand the sexual behavior of gender-diverse people, research is required among the personnel working in various community-led development program settings dealing with the welfare of such communities. Therefore, this study was conducted to assess the existing knowledge gaps regarding SSU among the community health mobilizers through a qualitative approach.

## 2. Materials and Methods

### 2.1. Study Site

This qualitative research was carried out in the month of July 2022 in the national capital territory (NCT) of Delhi in India.

### 2.2. Study Design and Gaining Access to the Study Subjects

An orientation and capacity-building workshop on “*Sexualized substance use in transgender people*” was organized for two days, in which working personnel of various community-led development program settings came from various parts of India. The workshop was a part of the Samarth 3 project. This opportunity was utilized to understand the thoughts of the participants and the community health mobilizers who agreed to participate in the research were considered for in-depth interviews (IDIs).

### 2.3. Participants and Eligibility Criteria

Community health mobilizers with the following inclusion criteria were enrolled: (a) those who agreed to participate and gave consent; (b) those with at least 10 years of work experience in the relevant field; and (c) those who were not abusing substances for the past one year.

### 2.4. Data Collection Tool

The IDIs were conducted at the start of the workshop following a brief description of the purpose, objective, and possible outcomes of this activity to the participants (Table 1). An experienced research investigator conducted one-on-one IDIs with each participant and recorded their responses. The probes were designed to cover a wide range of issues, including substance use, SSU, methods of substance use, overdose, and adverse case management, STIs in substance users, substance use in transgender people and their mental health, and multi-level stigma associated with SSU. IDIs were conducted in Hindi and English as per comprehension ability of the participants. Informed consent from all the participants was taken.

### 2.5. Data Analysis

Handwritten notes were used as raw data. The details of the participants, such as their age, gender, designation, geographical locations of their workplace, years of experience, and responses of the participants to probes were entered in Microsoft Excel. Normally distributed variables are presented as mean ± standard deviation, and non-normally distributed variables as median with interquartile range (IQR). The transcribed IDI responses were analyzed through thematic content analysis. The research findings were substantiated qualitatively by being transcribed verbatim. The verbatim transcripts were translated into English with pseudonyms and numerical codes to maintain confidentiality. A gap analysis was performed to determine in which areas the community health mobilizers lacked knowledge.

## 3. Results

### 3.1. Participant Demographics

**Figure 1:**
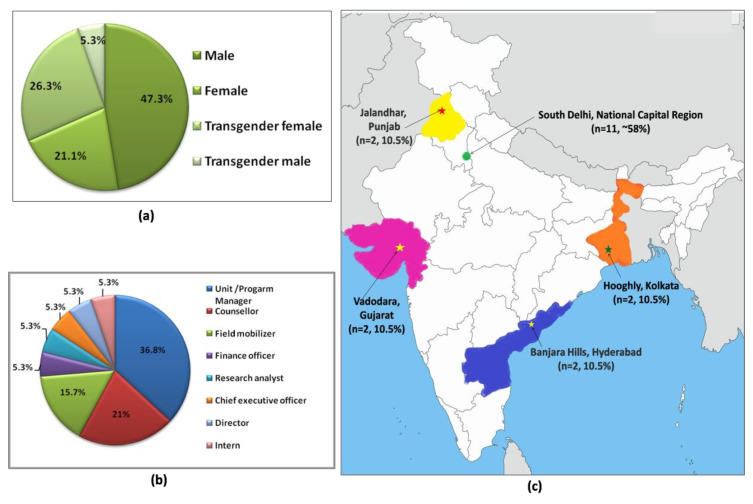
Classification of the participants based on (**a**) gender, (**b**) work profile, and (**c**) the geographical distribution of their organization.

A total of 19 community health mobilizers participated in the study, of which nine were men (47.4%), four were women (21.1%), five were trans females (26.3%), and one was trans male (5.2%) (Figure 1a). Their mean age was 36.4 ± 9.2 years and they had 13.3 ± 7.4 years of work experience (median 13 (IQR 10-18)). Out of the 19 health mobilizers, 37% were involved in managerial tasks, 21% in counselling, and 16% in field mobilization of sexualized substance users (Figure 1b). The participants were associated with one or more community led initiatives and groups majorly spread across five states of India (Figure 1c).

### 3.2. Thematic Content Analysis

**Figure 2:**
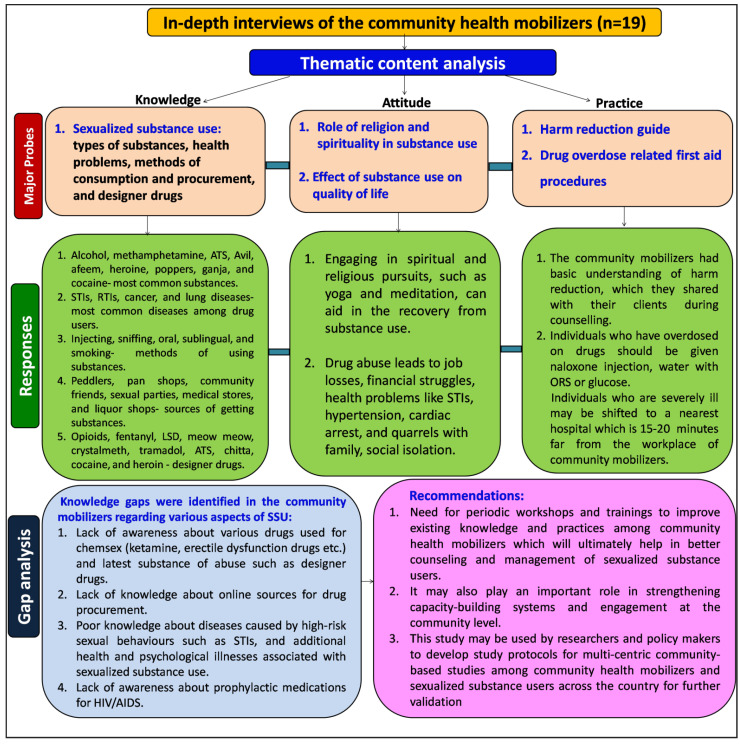
Overview of the study.

The IDIs were analyzed by a thematic content analysis approach. Three major themes emerged through their responses under which several sub-themes were identified. The observations and findings in the sections hereon are based on the themes of knowledge, attitudes, and practices of community health mobilizers working for the welfare of sexualized substance users (Figure 2).

#### 3.2.1. Participants’ Response to Knowledge-Assessment Probes

##### Sexualized Substance Use

(a) The participants were probed about the various substances that are used for sexual purposes which they had heard of or seen. Eleven of the participants (58%) answered alcohol, followed by M.D. (actual name 3,4-methylenedioxymethamphetamine MDMA; *n* = 7; 37%), and amphetamine type stimulants (ATS; *n* = 7; 37%).The participants’ responses were classified as follows: (i) central nervous system (CNS) depressants such as alcohol, MDMA, ATS, Avil, (ii) opioids such as afeem (opium), heroin, smack, (iii) inhalants such as poppers, (iv) cannabis such as ganja (marijuana), and (v) CNS stimulants such as cocaine.

(b) The participants were assessed for their knowledge of designer drugs. A “designer drug” is defined as a synthetic version of a controlled substance (such as heroin) that has a slightly altered molecular structure to evade restrictions against illegal substances [7]. The participants lacked knowledge about designer drugs and referred to the following substances as designer drugs: opioids, fentanyl, LSD, meow meow, crystal meth, tramadol, ATS, pregabalin, MD, white sugar, brown sugar, poppers, anabolic steroids, hallucinogens, chitta, cocaine, heroin, and synthetic drugs.

(c) The participants were questioned about the most common methods of consumption of substances they had heard from their clients. Most of them responded that injecting (*n* = 14; 74%), whether intravenously or intramuscularly, was the most common method of substance abuse, followed by inhaling (*n* = 11; 58%), and consuming orally (*n* = 11; 58%). Some of them also mentioned smoking and sublingual.

(d) The participants were assessed for their knowledge of various means for the procurement of sexualized substances, and they mentioned peddlers, pan shops, community friends, slums, liquor shops, border areas, pubs, medical stores, sexual parties, spas, hotels, rickshaw pullers, and sexual partners.

(e) According to the participants (*n* = 12; 63%), sexually transmitted infections were the most common diseases they had heard about or seen among substance users. Most of them believed HIV (*n* = 11; 58%) and viral hepatitis (hepatitis B, C infections; *n* = 10; 53%) were the most common STIs. Some of them had also observed reproductive tract infections (RTIs; *n* = 5; 26%) and other STIs such as syphilis, vaginal/cervical discharge syndrome (VCD), and urethral discharge (UD) among sexualized substance users.

##### Prophylaxis

(a) The participants were asked if they were familiar with the terms “pre-exposure prophylaxis or PrEP” and “post-exposure prophylaxis or PEP.” Twelve (63%) and thirteen participants (68%) have substantial knowledge about PrEP and PEP, respectively. The remaining participants was unaware of these terms.

##### Drug Overdose Related Complications

(a) The participants were questioned regarding the symptoms they observed in cases of drug overdose. They responded that people typically present with trembling, fear, hallucinations, nausea, vomiting, dehydration, hypertension, redness of the eyes, pinpoint pupils, seizures, loss of appetite, breathlessness, anxiety, forgetfulness, disorientation, illusion, mood swings, depression, lack of concentration, excessive drooling, yellowing of the skin, and hyperactivity. The severity of these symptoms varies depending on the level of intoxication.

##### Relapse to Addiction

(a) The participants were questioned if they had ever witnessed a relapse in substance users after quitting. Only a small number of participants (*n* = 2; 11%) claimed to have never witnessed a relapse after deaddiction. However, 89% of the participants stated that most of the cases would recur after six months to a year after initial treatment.

Treatment includes medications and devices that help to suppress withdrawal symptoms during detoxification. Detoxification is not in itself “treatment,” but only the first step in the process. Patients who do not receive any further treatment after detoxification usually resume their drug use [8].

Two participants stated that 90–100% of cases relapse after first treatment, while one participant stated that there are 50–70% of recurrence possibilities within 1, 3, 5, or 12 months of quitting. One participant claimed that, in his experience, relapses occurred in 2 out of every 10 cases (20%), while another claimed that relapses occurred in 6 out of every 25 cases (24%).

(b) They were also questioned regarding the potential triggers for relapse in substance users. They mentioned withdrawal syndrome, family problems, interpersonal troubles such as breakups, anxiety, and depression as the possible triggers.

“*People suffer from withdrawal syndrome after quitting drugs and so start abusing drugs again*”

—Bharat (CBO3)

“*Stress, peer pressure, and sex pleasure are some of the major triggers for relapse*”

—Vicky (CBO2)

“*Relationship, problems in family, office pressure, bullying in college, mind distraction are some of the reasons people start taking drugs again*”

—Shaurya (CBO4)

##### Work Challenges

(a) The participants were asked whether they had faced any legal challenges while working for the welfare of substance users. A few individuals experienced criticism and discrimination from their relatives and neighbors, but most of the participants said they did not encounter any legal obstacles.

“*Legal barriers not faced till date…. but dealt with structural stigma, people stigma, social stigma, and self-stigma*”

—Sunil (CBO1)

“*Sometimes police says that we are involved in substance sale*”

—Piku (CBO14)

(b) The participants were questioned about the reasons they heard from their clients that contributed to their substance abuse. They responded that their clients started using drugs because of peer pressure, to relieve stress, anxiety, and grief, for sexual pleasure, to counteract any feelings of guilt, and to overcome inhibition for sex work.

“*People usually start taking drugs out of curiosity, for enhancing sexual pleasure…. some people do drugs for countering their inhibition, to overcome their guilt feeling, some people do it just for fun*”

—Bharat (CBO3)

“*Common reasons which I have heard for doing drugs are to overcome depression after breakup …...People with high ambition take drugs to relieve stress and workload*”

—Barkha (CBO17)

“*First reason, people feel good after taking drugs, next they want to reduce their anxiety, and depression…. but it’s vice versa, like people who take drugs become prone to anxiety and depression because they lose their jobs, and their personal lives get jeopardized…. Genetics, and environment also contribute for drug abuse like peer group, hang out places etc*”

—Sunil (CBO1)

“*The reasons which I have heard from my clients are that…they lack confidence for doing sex with same sex…there is lack of confidence for standing on road for sexwork…some people take substance because they have entered to sex orgy parties*”

—Piku (CBO14)

##### Work Experience

(a) The participants were asked about the most typical age group they have dealt with during their time in service. Most of them responded that most of the drug users were between the ages of 16 and 40.

#### 3.2.2. Participants’ Response to Attitude-Assessment Probes

##### Role of Religion and Spirituality

(a) The participants were asked for their opinions on the relevance of religion and spirituality in helping people recover from drug abuse. Most of the participants (*n* = 14; 74%) held the view that engaging in spiritual and religious pursuits, such as yoga and meditation, can aid in the recovery from substance use. Few participants mentioned that many drug users visit “ojhas” (those who practice black magic), which they had heard during counseling.

“*Yes, most of the users go to quacks or ojhas (person who practice black magic)*”

—Ujjwal (CBO9)

“*If we can start meditation through spirituality, this will help to cope up from substance…some people go for meditation…some people start using if they get connected to religious group like religious akhada*”

—Piku (CBO14)

“*Sometimes it is possible but not always…. depends on client mental status*”

—Mayur (CBO6)

“*Many religion and spirituality things play a role in Punjab*”

—Bobby (CBO15)

##### Effect of Substance Use on Quality of Life

(a) The participants were questioned regarding how substance abuse affected the standard of living among their clients. According to them, substance abuse caused most of the users to lose their jobs. Their relationships with their parents, spouses, and friends suffered due to lack of concentration. They struggled financially because they became dependent on drugs and only craved for it. Many drug users started illegal activities, including smuggling, theft, and sex work, for quick cash to buy drugs.

“*Due to substance use, most of the time he/she lose job, they don’t feel seriousness about his/her lives…usually they lead their life very messy and most of time they don’t use condoms while engaging in sex work*”

—Sunil (CBO1)

#### 3.2.3. Participants’ Response to Practice-Assessment Probes

##### Harm Reduction

The participants were assessed for their knowledge of harm reduction. The community mobilizers understood harm reduction, which they shared with their clients during counselling. They advised their clients to (i) avoid unprotected sex; (ii) carry condoms and use them during sexual intercourse; (iv) go out with a friend who can monitor their behavior during drug consumption; (v) hand over their car keys to a responsible person before drinking alcohol and consuming drugs; (vi) stay hydrated and eat while drinking alcohol; (vii) take multivitamins [9]; and (vii) reduce the number of days they drink alcohol or consume drugs per week or month.

##### First-Aid for Drug Overdose

(a) The participants were asked if they were familiar with the procedures for administering first aid in the event of a drug overdose. They stated that they were aware of the airway, breathing, and cardiopulmonary resuscitation (ABC) method. Individuals who have overdosed on drugs are usually brought to them unconscious and dehydrated. Hence, they administer water, glucose, oral rehydration solution (ORS), and a naloxone injection (an opiate antidote) to such individuals. Individuals who are severely ill are referred and transported to a nearby hospital, which is 10–20 min away from their workplace.

“*In the event of a drug overdose, we first provide ABC therapy, second we call an ambulance, if we have naloxone injection then we give 2 mL dose to the patient and take him to the nearest hospital*”

—Bobby (CBO15)

“*We take the drug overdosed patients to the nearest government set up which is almost 20 min from our organization*”

—Bharat (CBO3)

### 3.3. Gap Analysis

**Figure 3:**
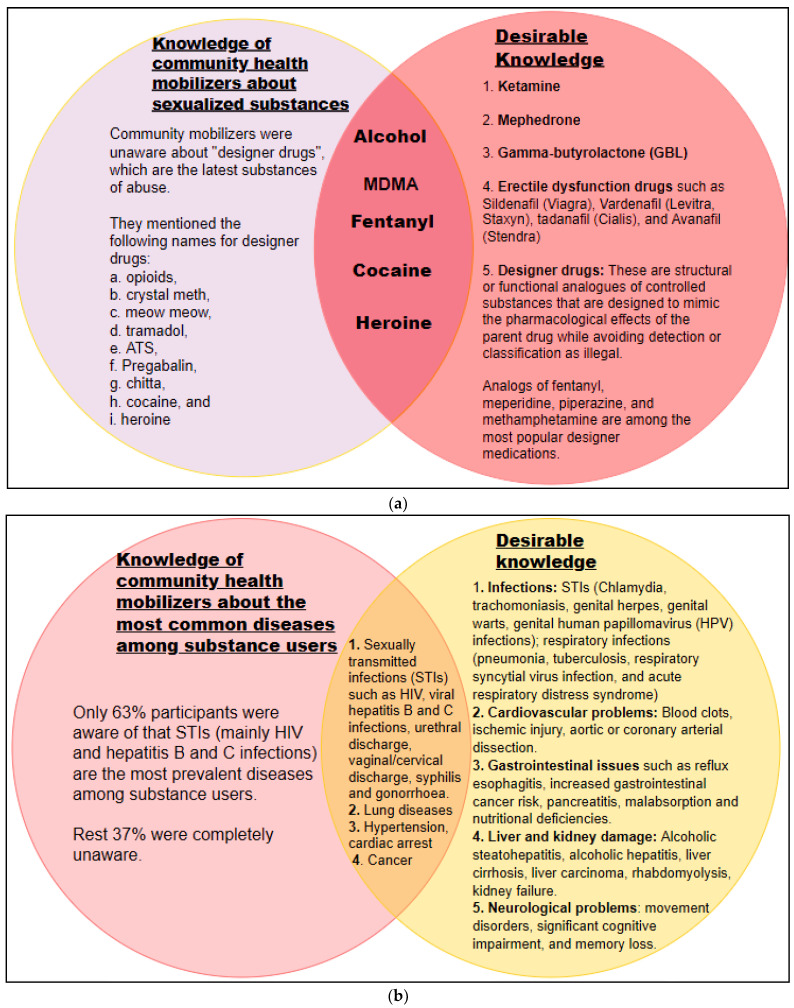

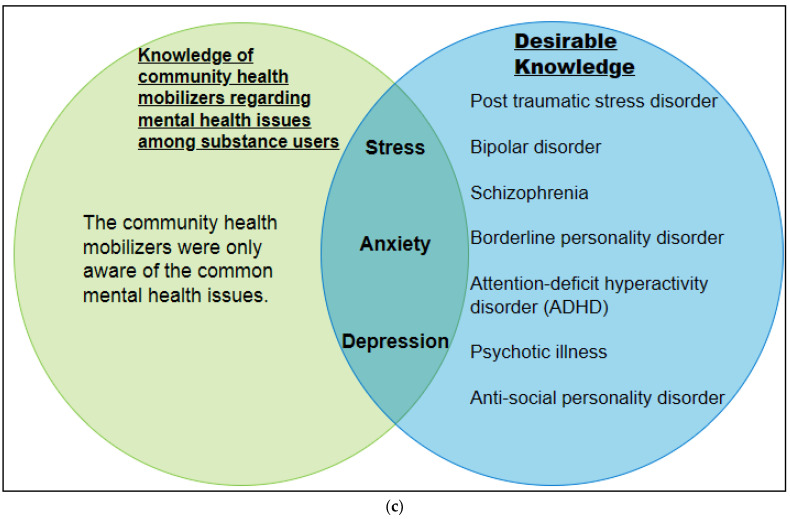
Venn diagrams showing major areas of knowledge gaps in community health mobilizers regarding (**a**) sexualize substances, (**b**) health problems and (**c**) psychological issues associated with sexualized substance use. The overlapping regions of the circles represent the desirable knowledge that the community health mobilizers possessed.

The study identified knowledge gaps in the following areas among community mobilizers:

(i) Sexualized substances: The participants were unaware about erectile dysfunction drugs, ketamine, and gamma-butyrolactone (GBL) which are very commonly used during chemsex. They were also unfamiliar with designer drugs, which are the latest sexualized substances of abuse (Figure 3a).

(ii) Drug procurement sources: The community mobilizers were unaware about the online sources such as mobile dating apps for purchasing drugs.

(iii) Health problems and psychological issues associated with SSU: Only 63% of participants had average-to-good knowledge of STIs such as HIV infection or acquired immunodeficiency syndrome (AIDS), viral hepatitis (hepatitis B and C infections), and mental health issues such as anxiety and depression that are common in sexualized substance users. However, they were unaware of the additional health and psychological issues associated with SSU (Figure 3b,c).

(iv) HIV prevention: Some of the community mobilizers (~30%) are unaware of PrEP and PEP, which are preventive medications for HIV/AIDS.

## 4. Discussion

This study was formative research in which the data on SSU was obtained through community health mobilizers. Through the responses of the participants, three major themes emerged, i.e., knowledge, attitudes, and practices of community heath mobilizers related to sexualized substance use.

The findings of this study are consistent with National Institute on Drug Abuse (NIDA) research, which found that substance use increases the risk of infectious disease transmission, such as HIV and viral hepatitis [10]. Intravenous substance use is the leading cause of virus transmission, accounting for approximately 6% of HIV diagnoses in 2015 [11]. Drug abuse can hasten the development of HIV and its consequences, especially on the brain. Clinical studies have revealed that, even in patients who are on antiretroviral therapy (ART), substance use may worsen AIDS-related mortality, accelerate disease progression, and increase viral loads [12]. Furthermore, people with substance use disorders are less likely to take HIV medication on a regular basis, worsening their situation [13,14,15].

Our results show consistency with Gonzalez-Baeza et al. (2018) who reported that SSU was associated with high-risk sexual behaviours and STIs in 742 HIV positive MSM. They have also reported that a total of 185 subjects (25%) had a diagnosis of depression, 175 (24%) an anxiety disorder, 57 (7.7%) a substance abuse disorder, and 9 (1.2%) previous psychotic episodes [16].

Our results show partial concordance with the study by Reback and Fletcher (2014), in which they examined substance use patterns among a group of 2136 transgender women. Their findings suggested that methamphetamine use was significantly higher among HIV positive women compared to HIV negative women (29.2% vs. 20.3%; *p* < 0. 001). Alcohol use was seen to be significantly higher in the HIV negative group indicating that methamphetamine use may be uniquely associated with increased sexual risk [17].

A study by D’Amico et al. (2020) found that the most common source of cannabis was recreational cannabis retailers (59.1%), followed by family or friends (51.5%), medical cannabis dispensaries (31.8%), and strangers or dealers (5.5%) [18]. In a study conducted by McCabe et al. (2019), high school students were asked from where they had procured prescription medications (such as opioids, stimulants, and anxiolytics) without a doctor’s prescription. The ten sources they have mentioned are as follows: acquired on the internet; taken without permission from a friend or relative; purchased from a friend or relative; obtained from one’s own past prescription; purchased from a drug dealer or stranger; and other means [19]. Hence, the responses of the participants showed partial similarity with these two studies.

The participants stated that most of the cases would recur after six months to a year of initial treatment/detoxification. Grau-Lopez et al., 2021, reported that 72.2% of their patients had relapsed after hospital detoxification at 6-month out-patient follow-up. Patients most likely to relapse were those dependent on opiates and those with compulsive use immediately prior to hospitalization [20].

The participants were well-aware of the specific symptoms and basic first-aid care in the event of a drug overdose. However, they were unaware of the health-complications associated with drug-overdose and classes/types of drugs responsible for complications. It has been previously reported that 15% of patients hospitalized with acute drug overdose suffer adverse cardiovascular events (ACVE) which require additional medical treatment, rehabilitation, and/or cause death [21]. The types of drugs associated with ACVE were class III antidysrhythmic, sodium channel blockers, antidepressants, and antipsychotics [22]. Jones et al., 2021 reported that patients who lose consciousness due to a drug overdose are at a higher risk for acute extremity compartment syndrome [23].

The participants responded that the substance users had a poor quality of life in terms of health, family, and employment. Treatments for chronic diseases (e.g., substance use disorder) are evaluated through subjective patient assessments on outcomes other than morbidity and mortality, and quality of life (QoL) is the most relevant measurement [24]. QoL measures add patients’ subjective assessments of the impacts that substance use disorder and its treatment can have on their lives [25]. Such measures privilege the patient in determining whether their employment status, health, and family contact are satisfactory or not. QoL measures may also help clinicians recognize problems other than the specifics of the disorder and hence make better treatment decisions and priorities [26,27].

In this study, the community health mobilizers had only basic knowledge of harm reduction. Harm reduction is defined as a set of practical strategies and ideas aimed at reducing negative consequences associated with drug use [28]. Harm reduction awareness and methods are lacking in India. In the United States, harm reduction programs were initially established to reduce the risk of infectious diseases, such as HIV, hepatitis C, and bacterial infections transmitted through nonsterile injection practices. Amid the HIV crisis in the 1980s and 1990s, syringe service programs provided sterile injection equipment and risk reduction counseling to people who inject drugs. As the risk for opioid overdose increased during the early 2000s, overdose education and naloxone distribution (OEND) programs also emerged [29]. Condom distribution and HIV pre- and post-exposure prophylaxis are the other harm reduction interventions. Additional harm reduction interventions include distribution of fentanyl test strips, which are used to detect fentanyl in drug samples and have been shown to be feasible and acceptable among young adults [30]. Similar strategies should be implemented in India.

The participants have faced criticism and discrimination from their family, relatives, and neighbors for working with sexualized substance users. Several studies reported that substance abuse counselors are at high risk for “burnout” due to low wages and a lack of prestige in their job [31,32,33]. Burnout is a term to describe aspects of (1) emotional exhaustion (inability to feel compassion for clients), (2) depersonalization (detachment from the emotional needs of their client), and (3) lack of personal accomplishment (critical evaluation of oneself) [34].

One of the many strengths of this study lies in inclusion of participants with at least ten years of work experience in the relevant field, i.e., counselling of substance users. The study consisted of mixed-group participants (males, females, and transgender persons). They were fluent in their respective local languages and were familiar with the local terminologies that people use to mention substances, e.g., ganja for marijuana, chitta for heroin (in Punjab), afeem for opium, and smack for black heroine. The study identified knowledge gaps in community health mobilizers.

The health mobilizers were unaware of different types of sexualized substances such as ketamine, mephedrone, GHB/GBL, erectile dysfunction drugs, and designer drugs. The usage of these psychoactive substances is common in MSM [35]. Designer drugs appeared in the early 2000s and participated in the development of chemsex [35]. The designer drugs are sold as bath salts “not for human consumption” to circumvent legislation [36]. They belong to the phenylethylamine family [37] and are psychostimulants with an amphetamine- or cocaine-like effect. They generate agitation, euphoria, and empathy in users and increase libido and sexual performance.

The participants were unaware of the online sources of drug procurement. The internet has played a significant role in the evolution of sexual behaviours, both in terms of sexual dating and the availability of new synthetic substances [35]. The practice of combining sex and psychotropic substance usage has increased steadily with the development of mobile applications. The introduction of geolocation applications in 1990 changed the way gay and bisexual men met [38] and contributed to the development of chemsex. These new applications enable users to meet one or more partners in their immediate surroundings and express their sexual desires and preferences through the practice of chemsex [39,40].

Only 63% of the participants had average-to-good knowledge of STIs. Chemsex has been linked to a significantly increased risk of contracting STIs and bloodborne viruses (BBVs) [35]. Hepatitis B, hepatitis C, syphilis, and HIV are more common in men who use methamphetamine and Viagra and/or who inject drugs intravenously [35,41].

The participants were also unaware of the complex health issues associated with SSU. In the study of Spiller et al. (2011) the main clinical effects (>10%) related to mephedrone consumption were agitation (82%), combative violent behavior (57%), tachycardia (56%), hallucinations (40%), paranoia (36%), confusion (34%), myoclonus (19%), hypertension (17%), chest pain (17%), and mydriasis (13%) [42]. It has been reported that regular GBL consumption alters cognitive functions, particularly memory and emotion management [43]. GBL overdose causes significant sleepiness and hypothermia that can lead to coma and death [35]. Ketamine can cause hallucinations at higher doses. Urologic complications such as ulcerative cystitis have been described with ketamine [44].

About 30% of the participants were unaware of PrEP and PEP. PrEP includes medications used to prevent HIV infection. It is extremely effective at preventing HIV when taken as prescribed. It reduces the risk of contracting HIV from sexual intercourse and from injected drug use. PEP includes HIV medications which should be taken within 72 h (3 days) of possible HIV exposure to prevent HIV infection and development of disease [45].

Therefore, this study suggested that the community health mobilizers only possess basic healthcare knowledge and lack “structural competency” in primary healthcare practice. Structural competence is defined as ‘the capacity for health professionals to recognize and respond to health and illness as the downstream effects of broad social, political, and economic structures [46].

The study has certain limitations. Only the community health mobilizers who agreed to participate were enrolled for the study. It might be possible that those who were willing to participate in the workshop were more knowledgeable than those not willing to participate. The participants belonged to only five states of India; therefore, geographic representation was limited. Their facts and assumptions could not be confirmed with sexualized substance users.

## 5. Conclusions

This study qualitatively assessed the knowledge, attitudes, and practices of community health mobilizers regarding SSU. In-depth interviews with the study participants revealed significant knowledge gaps such as identifying different sexualized substances, drug procurement, HIV prevention, and complex health and psychological issues associated with SSU. This implies the need for periodic workshops and trainings to improve existing knowledge and practices among community health mobilizers. It may also play an important role in strengthening capacity-building systems and engagement at the community level. Interactions between professionals, community representatives, and field mobilizers may provide scope for better challenge mapping at the community level, identification of issues at individual levels, mobilization towards health facilities, and counseling for better management of sexualized substance users. Based on the participants’ assessment of the compliance of the community towards the disseminated information, further counseling for safe sexual practices, substance use, and harm reduction may be provided. This study is formative research and the research priorities identified are based on the qualitative analysis and the knowledge gaps. This may help social scientists and policy makers to develop protocols for conducting multi-centric, community-based studies among community health mobilizers and sexualized substance users across the country.

## Figures and Tables

**Table 1:** Qualitative interview guide.

Domains	Enquiries	Probes
Sexualized substance use	What do you know about SSU?	Types of substances, recent substances of abuse such as designer drugs, methods of consumption and procurement, diseases, and infections
Prophylaxis	What do you know about prophylaxis for HIV/STI?	Pre-exposure prophylaxis (PrEP) and post-exposure prophylaxis (PEP)
Drug overdose related complications and first-aid procedures	How do you manage in case of drug overdose?	Symptoms of drug overdose, harm reduction, possible ways of getting services
Relapse to addiction	What do you know about relapse after deaddiction?	Rates of relapse, possible triggers
Work challenges	Would you like to share the problems you have faced while working with substance users?	Stigma, discrimination, legal challenges
Work experience	Would you like to share your various experiences working with substance users?	Age group, relationship with family members, role of religion and spirituality, effect on quality of life

## Data Availability

The datasets generated and/or analyzed during the current study are not publicly available. They are available from the corresponding author on reasonable request.
